# Female Pelvic Vein Embolization: Indications, Techniques, and Outcomes

**DOI:** 10.1007/s00270-015-1074-7

**Published:** 2015-03-25

**Authors:** Anthony James Lopez

**Affiliations:** The Imaging Clinic, Thursley Hall, Farnham Lane, Haslemere, Surrey GU27 1HA UK

**Keywords:** Venous intervention, Diagnostic, Embolization/embolotherapy, Pain management, Sclerotherapy, Sedation, Vein, Pain, Pelvic congestion syndrome/chronic pelvic pain, Varices, Varicocele, Varicose veins

## Abstract

Until recently, the main indication for pelvic vein embolization (PVE) in women was to treat pelvic venous congestion syndrome (PVC) but increasingly, patients with refluxing pelvic veins associated with leg varicosities are also being treated. A more unusual reason for PVE is to treat pelvic venous malformations, although such lesions may be treated with sclerotherapy alone. Embolotherapy for treating PVC has been performed for many years with several published studies included in this review, whilst an emerging indication for PVE is to treat lower limb varicosities associated with pelvic vein reflux. Neither group, however, has been subjected to an adequate randomized, controlled trial. Consequently, some of the information presented in this review should be considered anecdotal (level III evidence) at this stage, and a satisfactory ‘proof’ of clinical efficacy remains deficient until higher-level evidence is presented. Furthermore, a wide range of techniques not accepted by all are used, and some standardization will be required based on future mandatory prospective studies. Large studies have also clearly shown an unacceptably high recurrence rate of leg varicose veins following venous surgery. Furthermore, minimally or non-invasive imaging is now revealing that there is a refluxing pelvic venous source in a significant percentage of women with de novo leg varicose veins, and many more with recurrent varicosities. Considering that just over half the world’s population is female and a significant number of women not only have pelvic venous reflux, but also have associated leg varicosities, minimally invasive treatment of pelvic venous incompetence will become a common procedure.

## Introduction

Ovarian and other pelvic varices (such as in the distribution of the internal iliac veins) are not an infrequent finding in adult women, and particularly those who have previously had at least one pregnancy associated with a vaginal delivery or at least a significant trial of labour [1]. However, they are also well described in asymptomatic parous women [2].

Traditionally, such varices were diagnosed directly on clinical examination of vulval or perivulvar areas, or indirectly from a symptomatic history supporting the clinical diagnosis of ‘pelvic venous congestion syndrome’. However, more recently, it has become apparent that most of these varicosities can only be demonstrated adequately with non-invasive or minimally invasive imaging techniques. Some centres regard catheter venography as the optimum [3, 4] and others, transvaginal [5] or transperineal [6] duplex ultrasound scanning. In the author’s experience, transvaginal Doppler has not been widely adopted as it is very much operator dependent and difficult to reproduce and validate, and thus confined to a very few specialist centres. In contrast, venography is widely available, more objectively comparable, and has been used to ‘confirm’ refluxing pelvic veins suggested on a leg vein Doppler mapping study.

Pelvic venous incompetence is usually the underlying aetiology in the causation of pelvic varices and has been well known to be manifest as pelvic venous congestion syndrome [7] and usually reflects damage of pelvic vein valves during parturition [8], but it also rarely results from congenital venous stenosis or webs such as in May–Thurner syndrome [9], or acquired venous stenosis perhaps associated with iatrogenic or other trauma [10], tumour or deep venous thrombosis [11]. This well-recognized but poorly understood condition presents with a spectrum of symptoms including non-cyclic pelvic and sometimes abdominal pain for greater than six months duration, dyspareunia, dysmenorrhea, haemorrhoids, bladder irritability, and symptoms of irritable bowel syndrome although there are several others [7]. However, the diagnosis can only be made by excluding other causes of chronic pelvic pain such as pelvic inflammatory disease, endometriosis, adenomyosis, fibroids, and prolapse.

Over the last fifteen years, many venous clinics have formally evaluated all patients with Doppler ultrasound evidence of lower limb venous insufficiency and evidence of pelvic venous origin on this leg study, using pelvic venography, transvaginal or transperineal duplex sonography, and identified as many as 15–20 % of patients with lower limb varicosities partly or completely of pelvic origin, reflecting significant pelvic venous incompetence [5]. However, the percentage of such patients rises to up to 30 % [12] if they have recurrent varicose veins whether originally treated by conventional surgery or more contemporary minimally invasive endovenous procedures. Similarly, in Perrin’s study of 170 patients, pelvic vein reflux was present in approximately 17 % of patients with recurrent varices after surgery [13]. The relationship between pelvic venous incompetence and both PVC and lower limb varicosities, however, remains intuitive and to some extent empirical clearly, requiring further evidence, and ideally should be subjected to a randomized controlled trial.

## Radiological Anatomy

A detailed understanding of the relevant pelvic venous anatomy including common variants is essential in the minimally invasive management of pelvic venous incompetence using endovenous techniques.

Usually, blood in the left ovarian vein drains into the inferior vena cava (IVC) via the left renal vein, whilst the right ovarian vein is typically a direct tributary into the IVC at a similar level variably between T12 and L2. Occasionally, the left ovarian vein is also a direct tributary into the IVC. Of course, much of this work is based on anatomical dissection, but is gradually being correlated with non-invasive imaging techniques [14].

Valves occur within the main ovarian truncal veins but less so in the internal iliac veins [15]. Ahlberg et al.’s study of 84 post mortem cases [15] suggested that valves were more frequently absent in men than women, and in both sexes, valves were more frequently absent on the left side.

The internal iliac (hypogastric) venous plexus has a far more variable appearance although there are a number of typical patterns. The veins accompany the anterior and posterior divisions of the internal iliac arteries, and it is the tributaries of the anterior division which are of most interest in treating symptomatic pelvic venous reflux. The internal iliac vein typically drains into the common iliac vein by joining the ipsilateral external iliac vein.

**Fig. 1:**
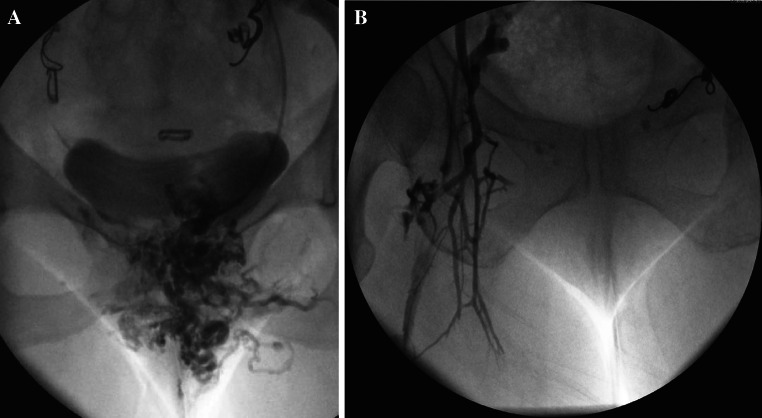
Refuxing internal iliac venous branches with vulval (**A**) and lower limb varicosities (**B**)

In the author’s experience, incompetent internal pudendal and broad ligament parametrial branches are most commonly associated with pelvic venous congestion syndrome *a priori*, whilst incompetent branches of the obturator and circumflex femoral veins are often associated with pelvic venous reflux into vulval (Fig. 1A) or lower limb varicosities (Fig. 1B). Incompetent ovarian (including round ligament) veins may contribute significantly to either clinical manifestation.

Varices are often identified not only in the uterine plexus with dilatation of the arcuate veins of Santorini in the uterine wall, but are also common in the vaginal wall, vulval veins, and periurethral and perianal veins. They are often linked to bladder irritability, urinary frequency, urge incontinence, and haemorrhoids.

**Fig. 2:**
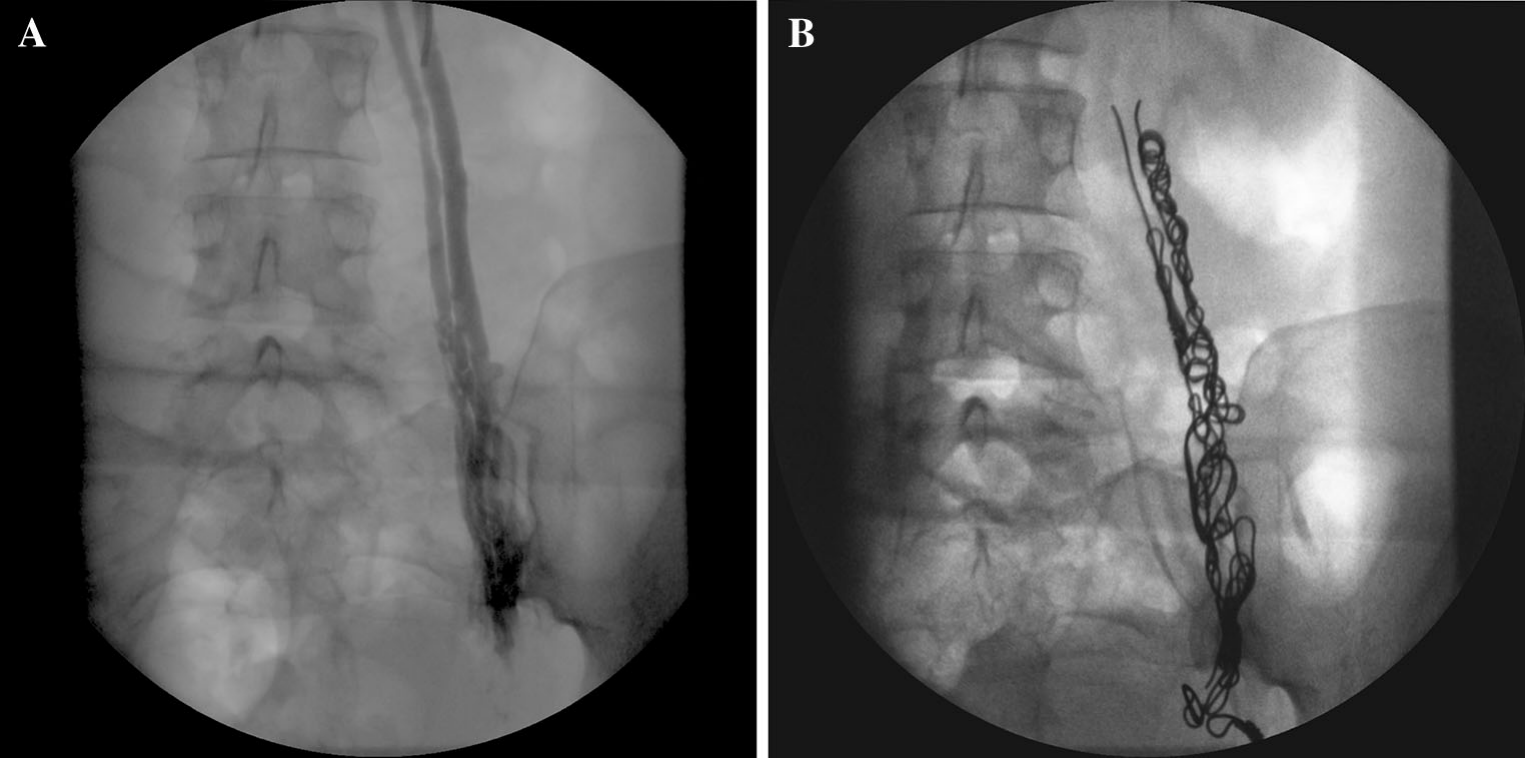
**A** and **B** Duplicated left ovarian vein before and after embolization

It is important to appreciate common and infrequent variants of normal anatomy. For example, the ovarian veins may be duplicated (Fig. 2), or they may drain into visceral branches such as the paravertebral or mesenteric veins [16], and clearly a failure to appreciate this can have disastrous consequences during therapeutic embolotherapy.

**Fig. 3:**
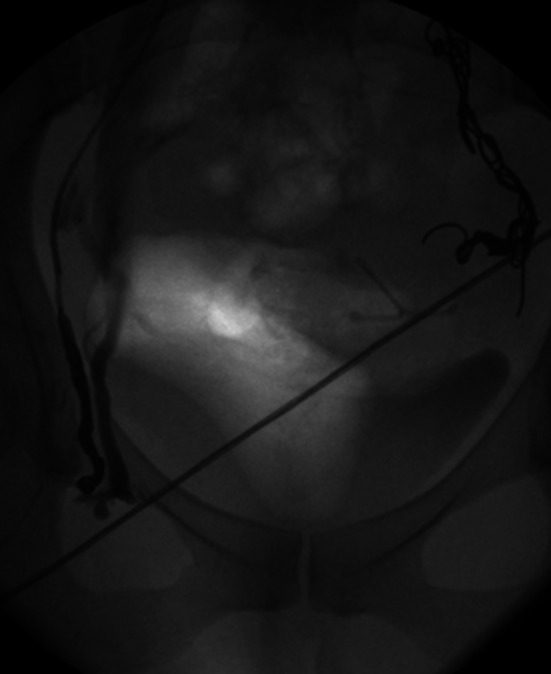
Obturator veins draining into both internal (catheter in situ) and external iliac veins (*right side*)

Similarly, the obturator vein which normally has two draining tributaries into the anterior division of the internal iliac vein may have two single draining veins into either, or both of, the internal and external iliac veins [17] (Fig. 3).

In the left side of the pelvis, the right common iliac artery may compress the left common iliac vein against the lumbar spine as it crosses it causing venous obstruction. This has been termed May–Thurner syndrome although more recently, the description “nonthrombotic iliac vein lesion” is increasingly used [18] and can involve both right and left iliac veins as well as other venous segments resulting in stasis of blood and the development of blood clots, typically with fibrous spurs.

**Fig. 4:**
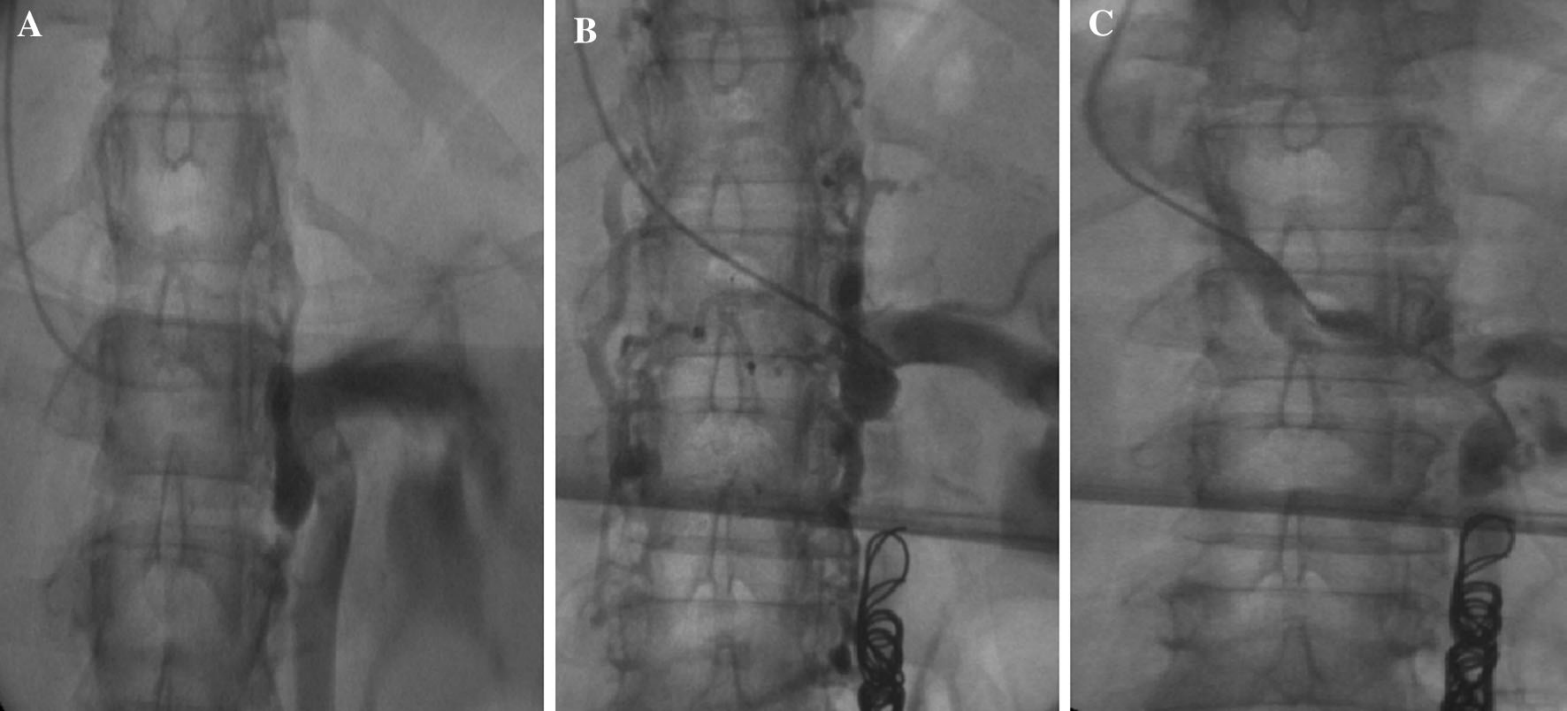
**A**–**C** Abdominal ‘nutcracker’ phenomenon—despite completely embolizing the enlarged left ovarian vein, the ‘compressed’ left renal vein still fills poorly on direct injection

A well-recognized anomaly is the abdominal ‘nutcracker’ phenomenon where the left renal vein is compressed by the overarching superior mesenteric artery as it traverses the retroperitoneum anterior to the lumbar spine to drain into the IVC [19] (Fig. 4). However, in the author’s opinion, this is an overstated cause of a left ovarian varicocele, symptomatic or otherwise.

The diagnosis should be suspected when it is difficult to traverse the left renal vein from the IVC especially if a reverse curve catheter is ultimately required (from a transjugular approach) to catheterize the more distal left renal vein. The appearance of collateral veins from the renal vein on venography or rapid reversal of flow into the ovarian vein (Fig. 4A) favours severe compression. Alternatively, a renocaval pressure gradient of >4 mmHg is commonly used to diagnose this entity, and some authors have stented the left renal vein for the condition in isolation or following ovarian vein embolization [20]. This may also improve renal venous drainage as in this case, where ovarian vein embolization may result in an elevated renal venous pressure.

As interest in pelvic venous insufficiency associated with pelvic venous reflux has developed alongside technological advances in non- and minimally invasive imaging techniques, attempts have been made to diagnose refluxing veins based on their diameters. For example, a truncal ovarian diameter of greater than 10 mm at its widest point has been suggested as indicative of reflux [17]. The same authors used other criteria including (1) uterine venous engorgement, (2) moderate or severe engorgement of the ovarian plexus, and (3) filling of the veins across the midline or filling of vulvar or thigh varicosities [17].

Others have used different criteria. For example, Asciutto et al. [21] used the following:

- Varicose reflux towards the ipsi- or contralateral proximal thigh
- Visualization of ‘reflux’ throughout the entire course of the ovarian vein
- Retrograde filling of the main stem of the internal iliac vein and at least one side branch (gluteal, ischial or obturator veins)
- Retrograde filling of contrast medium across the midline.

A number of studies have shown the incidence of refluxing left ovarian and bilateral internal iliac veins as fairly equal and together the commonest pattern [21–26, 27], and although it does not appear to predispose to a particular pattern of symptoms compared to involvement of other pelvic veins, the importance of treating these veins is increasingly being recognized [21, 27].

**Fig. 5:**
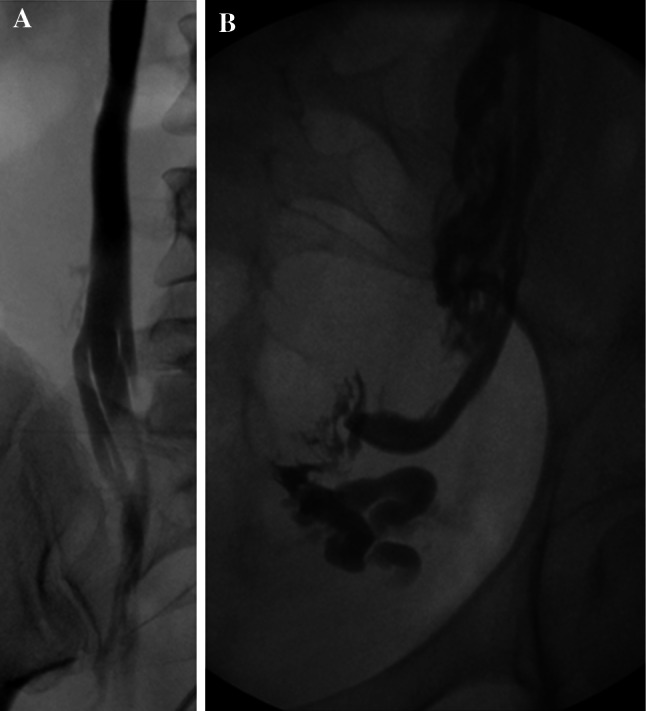
**A** and **B** Refluxing right and left ovarian veins

**Fig. 6:**
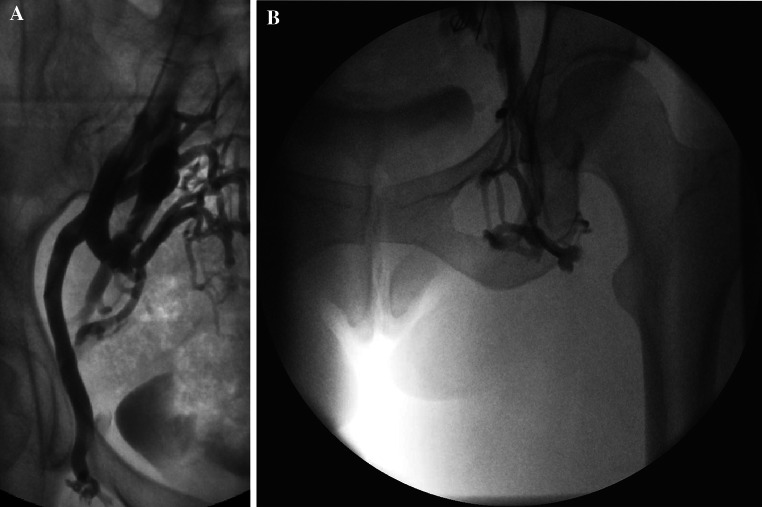
**A** and **B** Refluxing right and left obturator veins

Typical refluxing vein appearances using these criteria are as follows (Figs. 5A, B and 6A, B).

**Fig. 7:**
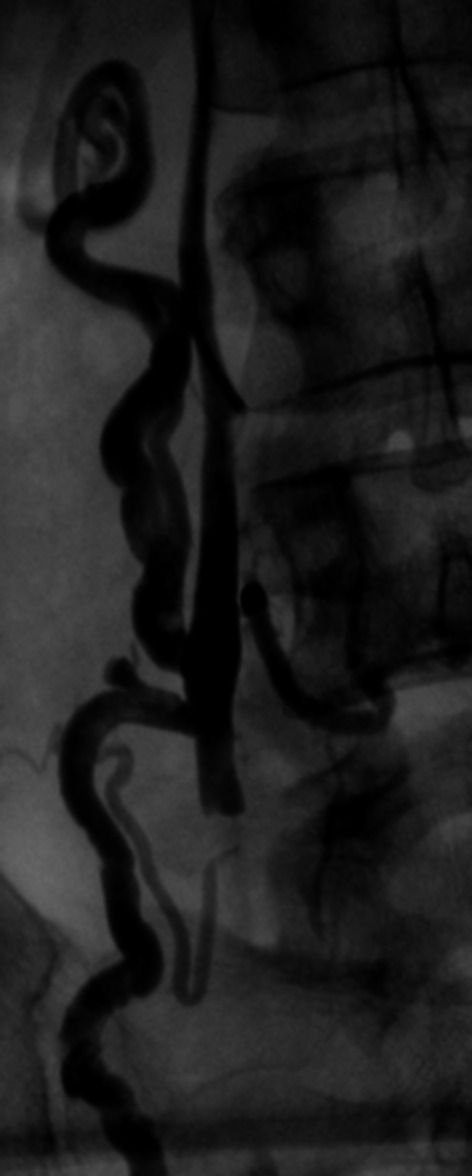
Non-refuxing ‘large’ right ovarian vein. Note ‘competent’ valve along main trunk and aberrant retroperitoneal branches

It is important to remember that large diameter veins do not always reflux and small apparently inconsequential veins may reflux, and it should be recognized that otherwise competent main truncal ovarian veins can be rendered ‘functionally’ incompetent by large incompetent perirenal or retroperitoneal veins (Fig. 7) where in this case, transvaginal duplex sonography failed to appreciate that this branch is significantly refluxing.

**Fig. 8:**
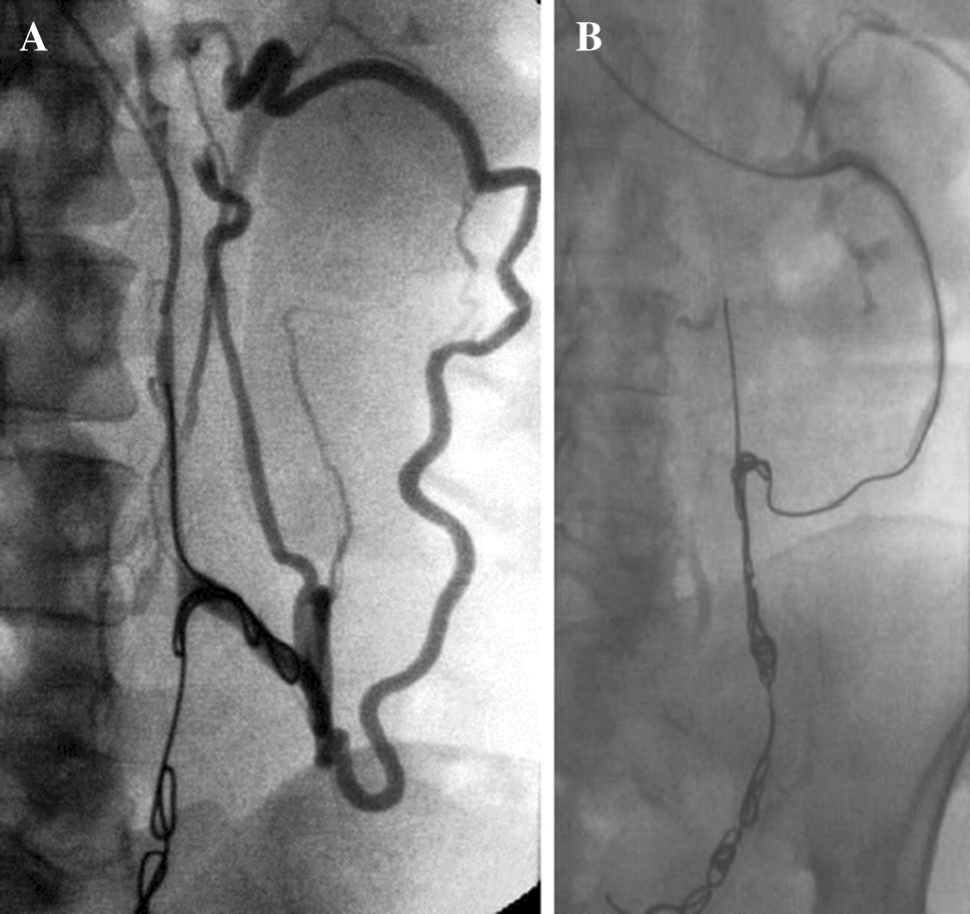
**A** and **B** Aberrant retroperitoneal veins before and after embolization

It is not at all uncommon to visualize large retroperitoneal aberrant veins draining into the gonadal veins, and most commonly the ovarian veins (Fig. 8), and these too need to be embolized to eliminate reflux and reduce recurrence.

Although representing less than 0.1 % of patients in most venous practices, male patients with pelvic venous reflux associated with lower limb insufficiency can be treated identically to females with pre-procedural assessment using either transrectal/transperineal duplex sonography, pelvic venography or ‘upright MRI’ to direct therapeutic embolization.

## Technical Considerations

One of the first documented reports of catheter embolization of incompetent ovarian veins was by Edwards et al. [16] in 1993. Several groups have subsequently reported series of patients [28–31] but with relatively low numbers and predominantly treating ovarian veins alone. More recently, a number of groups have started to treat the internal iliac veins also [22, 23] with the largest published series to date being reported by Kim et al. [24] who treated 131 patients aggressively; Laborda et al. [25] who treated 202 patients; and Monedero et al. [26] who treated 215 patients with recurrent varices after surgery, all using catheter venography as the basis of embolization.

This has now become a routine procedure in many centres, and the author has performed over 1000 such procedures with only very few complications limited to coil migration, minor neck bruising and neck/chest aching [32] on the post-procedural questionnaire delivered at the outpatient clinic review. Specifically, no pneumothorax was recorded although routine chest films were not performed and, whilst pelvic discomfort related to embolized veins is to be expected, it is always without adverse consequences requiring nothing more than simple analgesia for symptomatic relief.

The procedure is essentially the same regardless of the cause of pelvic venous incompetence, treatment typically being directed to the incompetent veins determined by the pre-procedural diagnostic modality used.

A number of approaches to the central visceral abdominal and pelvic veins are available including the femoral, subclavian and brachial veins although in the author’s experience, the transjugular or subclavian routes offer the most reliable access to all major relevant pelvic veins from a single puncture site.

The right internal jugular vein is felt by the author to be the most optimum and direct route in his considerable experience. It is highly visible and easy to access under ultrasound guidance, does not typically experience spasm like the brachial vein and affords an antegrade approach, i.e. essentially ‘downhill’, to all the relevant pelvic veins. There is also less requirement to reform angiographic catheters or use ‘reverse curve’ catheters such as a 4F Sos omni (Cook Inc, Bloomington, Indianapolis) or 5F Sim ‘sidewinder’ (Cordis, Miami Lakes, Florida), where there is less control of the embolic agents, as ‘turning’ a catheter in the opposite direction after pushing a catheter cranially from the groin makes it inherently unstable.

Embolization is typically performed in our institution under mild sedoanalgesia utilizing an opiate and a benzodiazepine together with an antiemetic, although there is no absolute requirement for ‘conscious sedation’ as the procedure is well tolerated. Typically, the right internal jugular vein (or external if internal unavailable) is directly punctured under ultrasound guidance using local anaesthesia.

‘Standard’ conventional catheters and guidewires are utilized typically with a 0.035” ‘system’ (rather than a 0.018” microcatheter ‘system’ placed co-axially), as the target veins are tortuous and dilated easily accommodating standard equipment. Typically, a 6F vascular sheath (Cordis, Miami Lakes, Florida), a 5F multipurpose angiographic catheter (Cordis, Miami Lakes, Florida), standard ‘moving core’ J 0.035” guidewire and an angled hydrophilic wire (Radiofocus, Terumo, Europe) are all that are required to complete a case in usually less than 45 min for up to four pelvic truncal veins.

Occasionally, a reverse curve catheter such as a 5F Sim catheter (Cordis, Miami Lakes, Florida) reformed at the common iliac venous confluence may be useful to catheterize a spastic or ‘difficult’ left renal vein (e.g. ‘nutcracker’ syndrome) in order to superselectively catheterize the left ovarian vein (Fig. 4), prior to reverting to a conventional catheter.

Although only a 5F catheter is required for embolization, it is important to remember that a 6F sheath will be necessary to enable a misplaced or displaced coil to be retrieved using an Amplatz Goose Neck snare (ev3 Inc, North Plymouth, Minnesota) or other retrieval device.

In contrast to conventional ‘open’ or laparoscopic surgical techniques, endovenous procedures use a minimally invasive approach to occlude and ultimately ablate refluxing veins [33]. However, physical media such as extreme heat (e.g. radiofrequency, laser, steam) used in treating refluxing ‘peripheral’ veins are inadvisable for use in the abdomen or pelvis given the proximity of visceral structures such as bowel and ureter, which may be irreversibly damaged.

The commonest embolic agents utilized are platinum embolization coils, foam, glue and liquid sclerosants [e.g. Polidocanol, 3 % sodium tetradecyl sulphate (STS)]. Platinum coils are MRI-compatible up to 1.5T, associated with little artefact on MRI (although considerably more on CT) and are not detected by an airport scanner which would be a nuisance to the patient and the airport agency. However, newer coils are being developed which also show little or no artefact on CT scanning.

These agents may be used in isolation or together. Indeed recently, STS has been used in association with a mechanical device used to disrupt the endothelium in the Clarivein system [34] and could potentially be used in pelvic veins, although compression of the ablated vein is integral to its efficacy and clearly this is difficult for pelvic veins. Similarly, glue used either alone or in the Sapheon system [35] could potentially be adopted for use in treating pelvic venous incompetence. However, regardless of the injurious agent used, it is often recommended that a foreign body be left in situ to obviate recanalization, and embolization coils are used by many authors to achieve this. Until more work has been completed and greater long-term outcomes assessed, this potentially makes use of the two latter systems financially unviable for now. Regardless of the agent used, higher-quality evidence in the form of properly conducted randomized controlled studies using a variety of currently available embolics and/or sclerosants is essential in progressing our understanding in this developing field, and these remain lacking.

In our institution, Spirale platinum embolization coils measuring between 8 and 16 mm in unconstrained diameters (BALT Extrusion, Montmorency, France) were initially used. More recently, 8–12-mm diameter coils ‘fibred’ with synthetic dacron (Cook Inc, Bloomington, Indianapolis), rather than plain non-fibred coils have been utilized as they achieve more rapid occlusion with fewer coils required, offer easier retrievability if misplaced enabling a lower procedural time and reduced exposure to ionizing radiation. Detachable coils may be particularly useful for the inexperienced operator embolizing in particular, the internal iliac branches and most proximal ovarian veins.

Techniques for coil deployment vary. In contrast to arterial embolization, where tight packing of coils may be advantageous to occlude flow at arterial pressure, it is the author’s opinion that a foreign body of appropriate size along near the entire course of the target vessel is all that is required in veins where flow is slow, and thrombosis almost inevitable. Furthermore, densely packing coils in these very long venous segments would be prohibitively expensive, as would the use of Amplatzer vascular plugs (AGA Medical Corporation, North Plymouth, Minnesota).

**Fig. 9:**
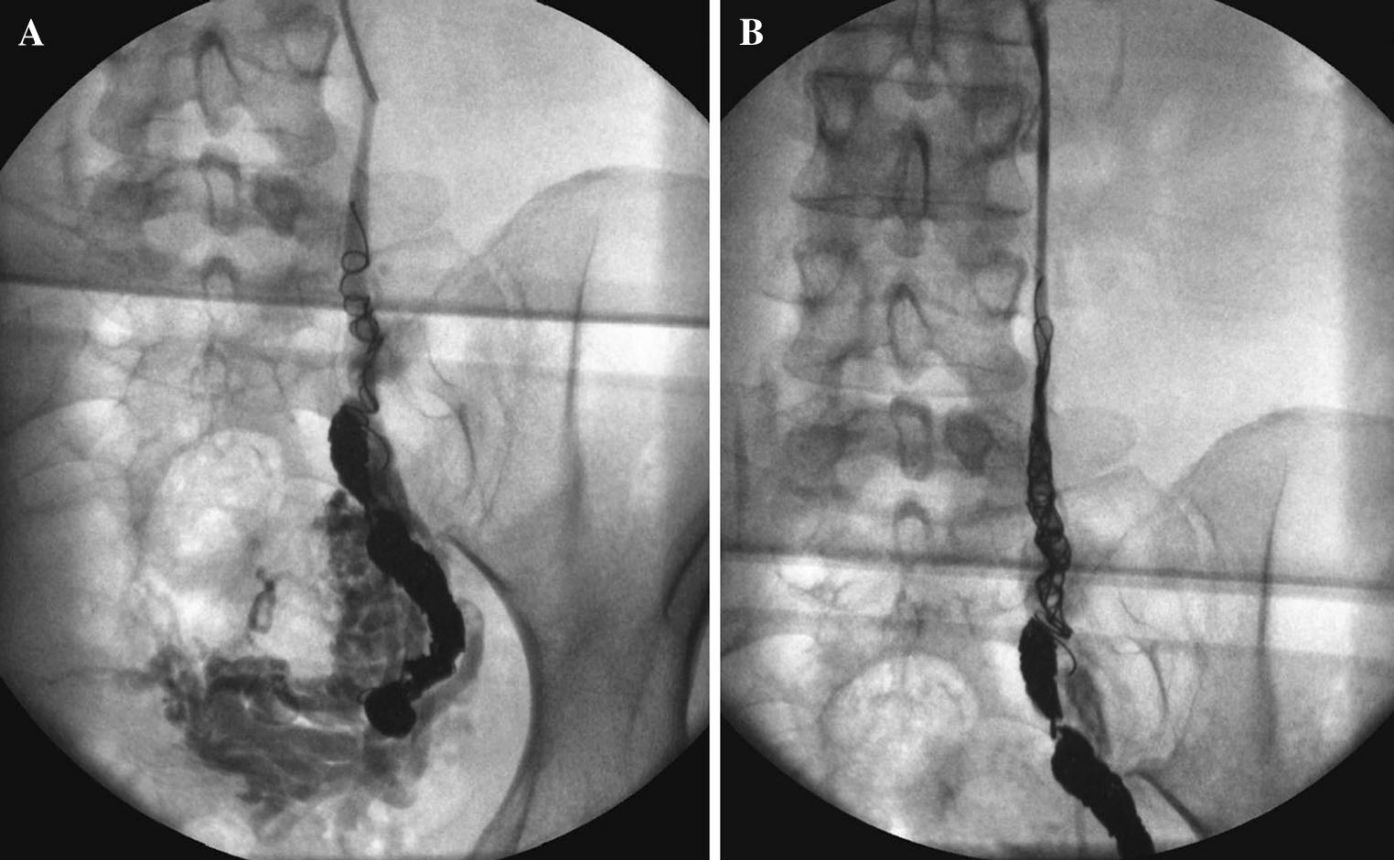
Persisting ovarian vein reflux following incomplete coil embolization (**A**) re-treated with ‘distal’ foam sclerotherapy and ‘completion’ coil embolization (**B**)

A fibred platinum coil is progressively ‘unwound’ along the vessel to achieve complete occlusion. The author feels it important to embolize the entire length of the incompetent vessels including their larger truncal draining tributaries to prevent new collateralization. Although there is no substantial evidence to support this, in the author’s experience, ‘failed’ interventions subsequently referred to a specialist unit are typically shown to have inadequate ‘coverage’ by coils (Fig. 9), and other experienced operators have adopted a similar strategy [24]. The process may be hastened by oversizing the coil diameter to vessel size and by instilling foam or a liquid embolic alongside or between coils.

**Fig. 10:**
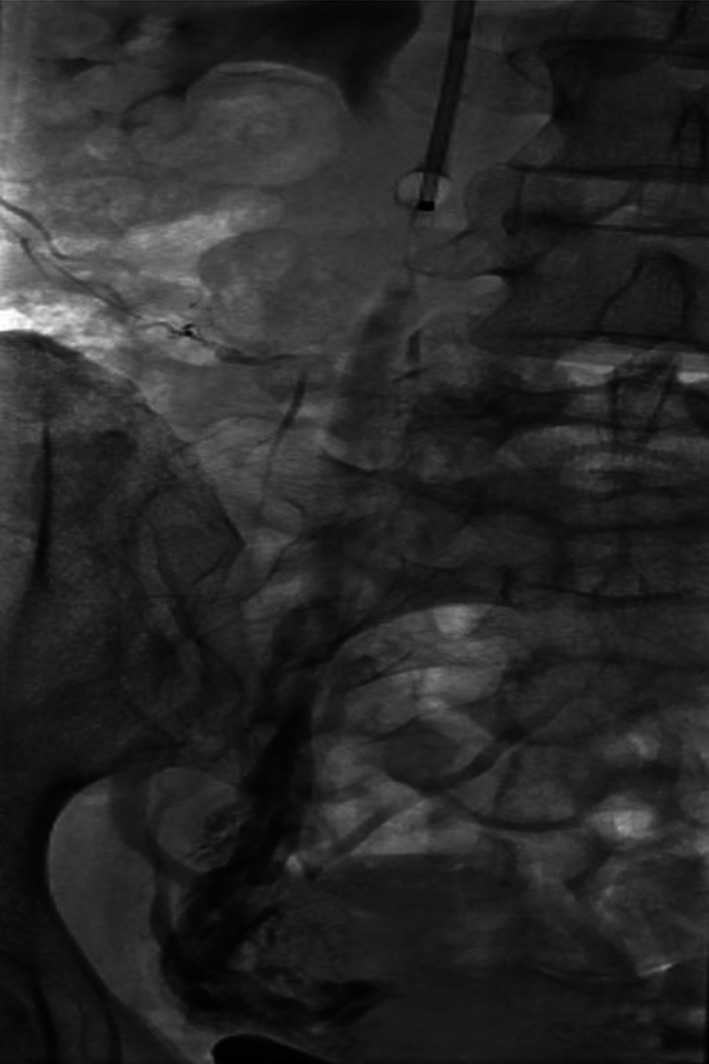
Foam sclerotherapy of right ovarian vein

Alternatively foam can be administered by free hand injection, or ‘controlled’ by using a balloon occlusion angiographic catheter (Fig. 10) in both cases by ‘pre-filling’ the catheter and target vein with iodinated contrast and injecting the foam under modest constant positive pressure to simply displace the contrast from the distal target vessel ensuring complete vessel coverage and minimal reflux. In this respect, it is best to use a O_2_/CO_2_ mixture with 3 % sodium tetradecyl sulphate in a 5:2 mixture [36] avoiding room air (which contains 80 % nitrogen increasing the theoretical risk of stroke) in the Trendelenburg position.

**Fig. 11:**
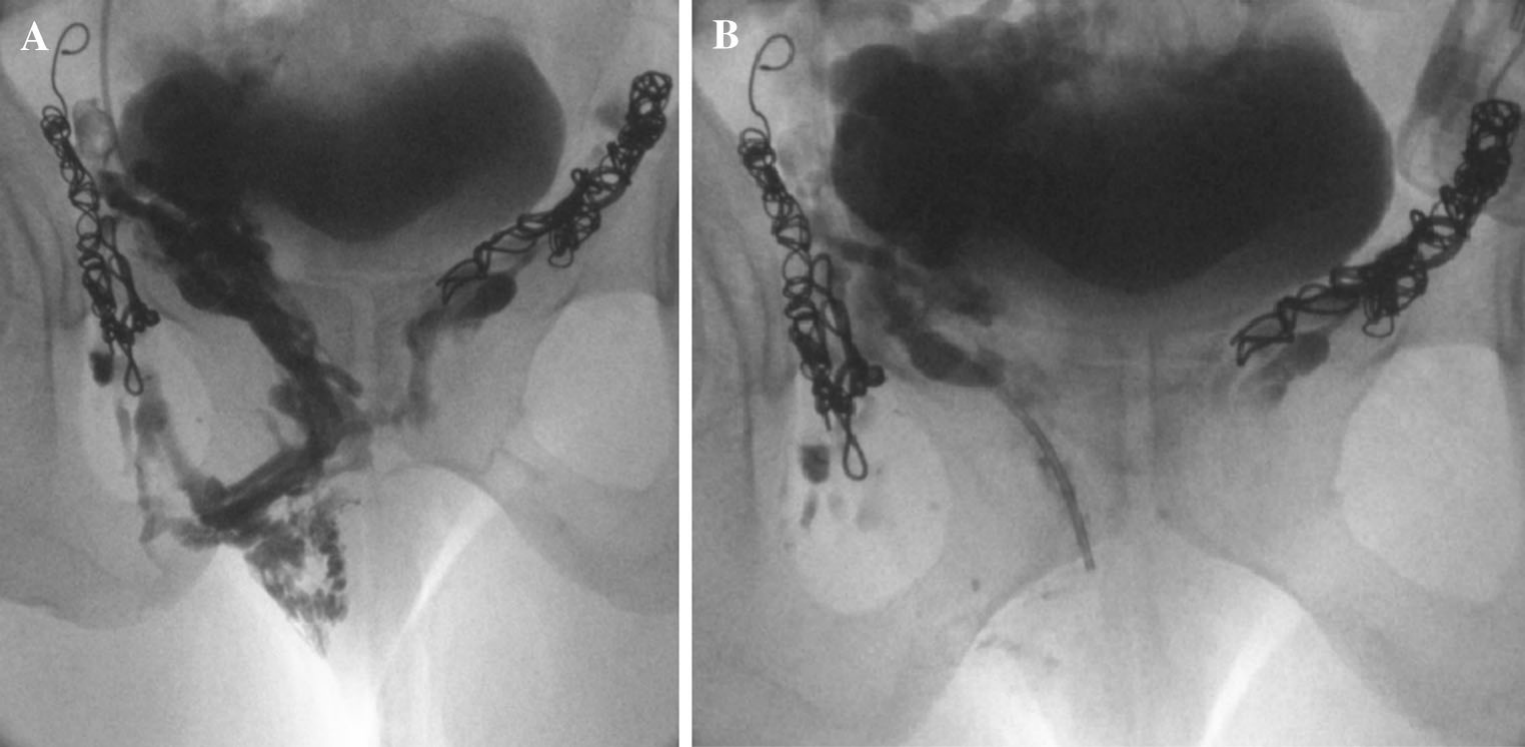
**A** and **B** Vulval varices treated with foam sclerotherapy and coil embolization

Foam is particularly good for treating the smallest ‘peripheral’ vulval and haemorrhoidal veins (Fig. 11).

**Fig. 12:**
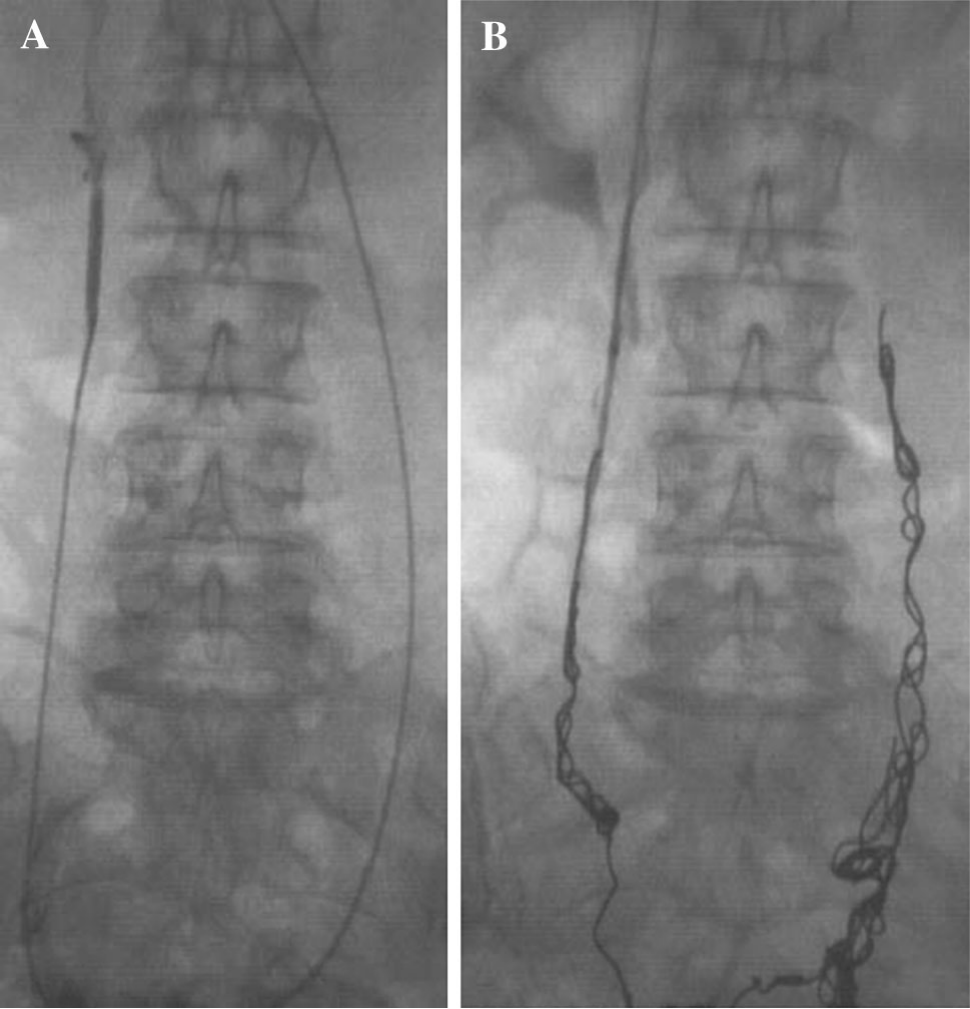
**A** and **B** Cross embolization of right ovarian vein from left vein

**Fig. 13:**
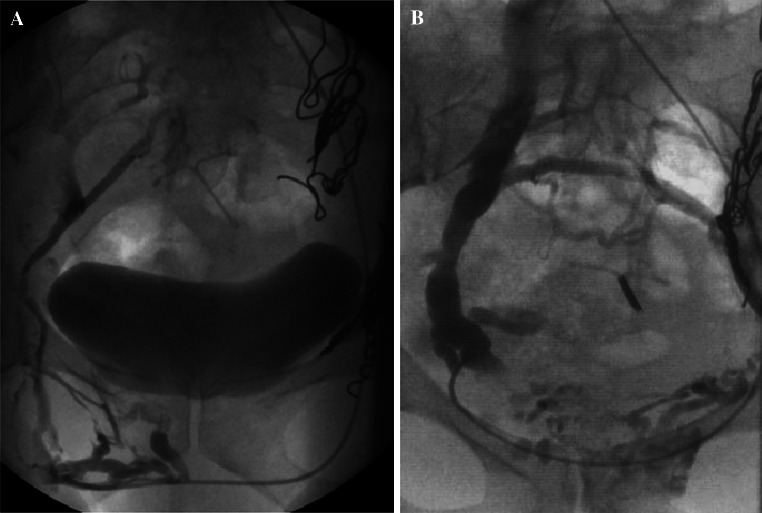
**A** and **B** Cross embolization of right vulval veins from left internal iliac vein

The rich venous plexus sometimes allows for contralateral catheterization and embolization, e.g. embolization of the right ovarian vein from the left ovarian vein (Fig. 12) or vice versa and embolization of the left internal iliac gonadal venous branches from its counterpart or vice versa (Fig. 13).

**Fig. 14:**
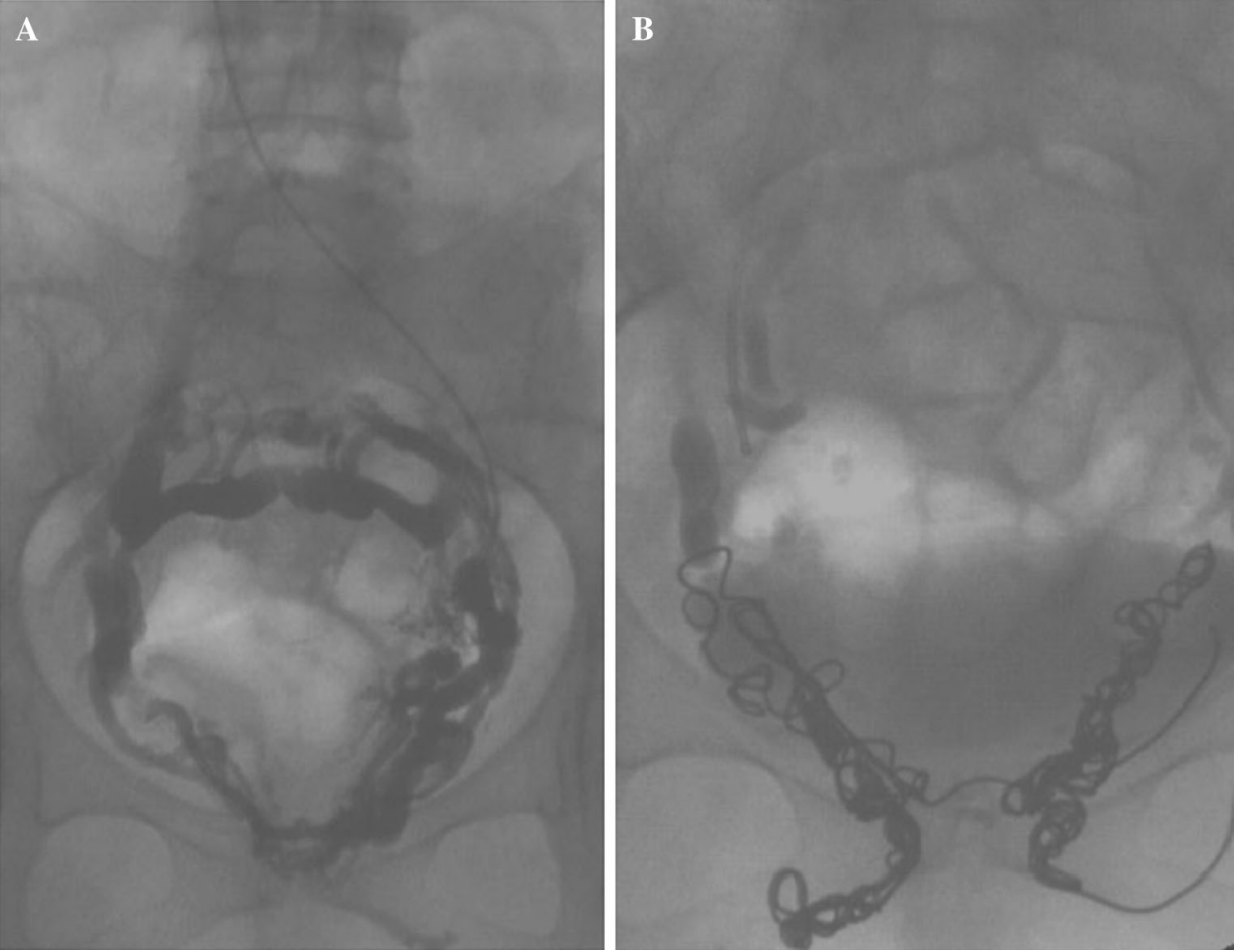
Pudendal and broad ligament venous embolization

For pelvic venous congestion, it is usual to catheterize refluxing pudendal or broad ligament branches (Fig. 14).

Patients are usually able to leave the hospital or clinic as soon as they have recovered from the sedation (if administered) and would expect to be discharged within 1–2 h. A consistent finding is pelvic aching and occasional discomfort at the needle puncture site, and can usually be successfully managed with a non-steroidal anti-inflammatory drug with only very rarely a requirement for stronger medication.

Immediately following embolization, the patient may experience mild-to-moderate discomfort for up to 5 days (but often shorter and rarely longer), and this typically responds rapidly to non-steroidal anti-inflammatory medication. In the author’s experience, there is never a requirement for patient-controlled analgesia (PCA).

After approximately 6 weeks, all patients have a follow-up Doppler ultrasound scan. If reflux is completely eliminated or there is very modest ‘trickle’ reflux, the patient is booked for a definitive endovenous leg vein procedure, e.g. laser treatment (EVLT), radiofrequency ablation (VNUS), or discharged if treated for PVC.

If more substantial persisting or new reflux is demonstrated, localized transcutaneous pelvic foam sclerotherapy can be performed [7] as foam is now less likely to pass cranially into the ‘central’ veins in significant volumes as the ‘back door’ to previously identified refluxing veins (but not necessarily new sources) has been virtually closed by pelvic truncal vein occlusion. However, if there is more substantial reflux perhaps through ‘undertreatment’, or new refluxing veins have opened up (more likely if the patient has an intervening pregnancy), further pelvic embolization may be indicated.

## Radiation Protection and Dosimetry

It is a fundamental principle to limit the patient and staff to as low a dose of ionizing radiation as is reasonably achievable, and it is mandatory to ensure that any benefit conferred by the technique outweighs the small but defined risk associated with the stochastic effects of ionizing radiation.

Modern interventional fluoroscopy equipment intensifies the image and uses a relatively low dose of radiation. The dose can be kept to a minimum using low milliamperage (mA) fluoroscopic screening and carefully controlling screening times. This allows perfectly adequate pictures for guidance during treatment but not “ultrasharp” imaging, which is perfectly acceptable, as a balance needs to be achieved. An analogy would be to use a 3 megapixel digital camera providing very affordable yet perfectly satisfactory pictures compared to a markedly expensive 15 megapixel camera producing sharper images!

The dose can be further reduced by limiting the exposure of body area to ionizing radiation with collimators (‘coning’), and minimizing the distance between the anode source of radiation and the patient. Clearly, the fewer the veins to be treated, the slimmer the patient and the less the complexity of the anatomy, the lower the radiation dose. Also, the more experienced the staff, the lower the procedure time and therefore the dose. In our institution, medical physicists have calculated the radiation dose during the procedure by measuring the ‘dose area product’ (DAP) and used the Monte Carlo software to yield an “effective dose”. The mean of these measurements (considering the number of veins embolized and given the degree of difficulty varies between patients) is 5200 cGy/cm^2^ which converts to an effective dose of approximately 6 mSv.

What does this mean in ‘real’ terms? This is equivalent to a CT scan of the abdomen and pelvis using a modern 128 multislice CT scanner at the same hospital. Alternatively, it equates to 3 years of ‘natural’ background radiation—we are all exposed to background radiation from the surroundings including the sun—(or 1 year of background radiation in some parts of the West of England, e.g. Cornwall). For UK patients, an alternative equivalent would be a return flight to Paris. The conversion is 0.0011 mSv per cGycm^2^, i.e. multiply the DAP by 0.0011 to get the effective dose (NB it will vary with the size of the patient and the software assumes rectangular collimation rather than the near circular image intensifier field).

## Complications

Complications are exceedingly rare with meticulous attention to the initial venous puncture and embolization technique. ‘Immediate’ complications include:

- Those related to drug administration, e.g. narcosis, sedation or rarely hypersensitivity, for example to iodinated contrast (reported in less than 1 % of patients with non-ionic contrast) [37].
- Venous puncture related, e.g. haematoma, pneumothorax (for venous catheterization via a neck vein)
- Embolization of non-target vessels, i.e. coil misplacement, e.g. left ovarian vein involving left renal vein, obturator or circumflex coil protruding into left external iliac or common femoral vein [38]. Caution is advised with liquid embolic agents as communications have been shown to exist between the ovarian veins and paravertebral veins and specifically between the left ovarian vein and splenic, ureteric and inferior mesenteric veins [16, 39].
- Stroke related to paradoxical emboli from coil migration or uncontrolled foam (most likely when using ‘room air’)

Delayed complications can include

**Fig. 15:**
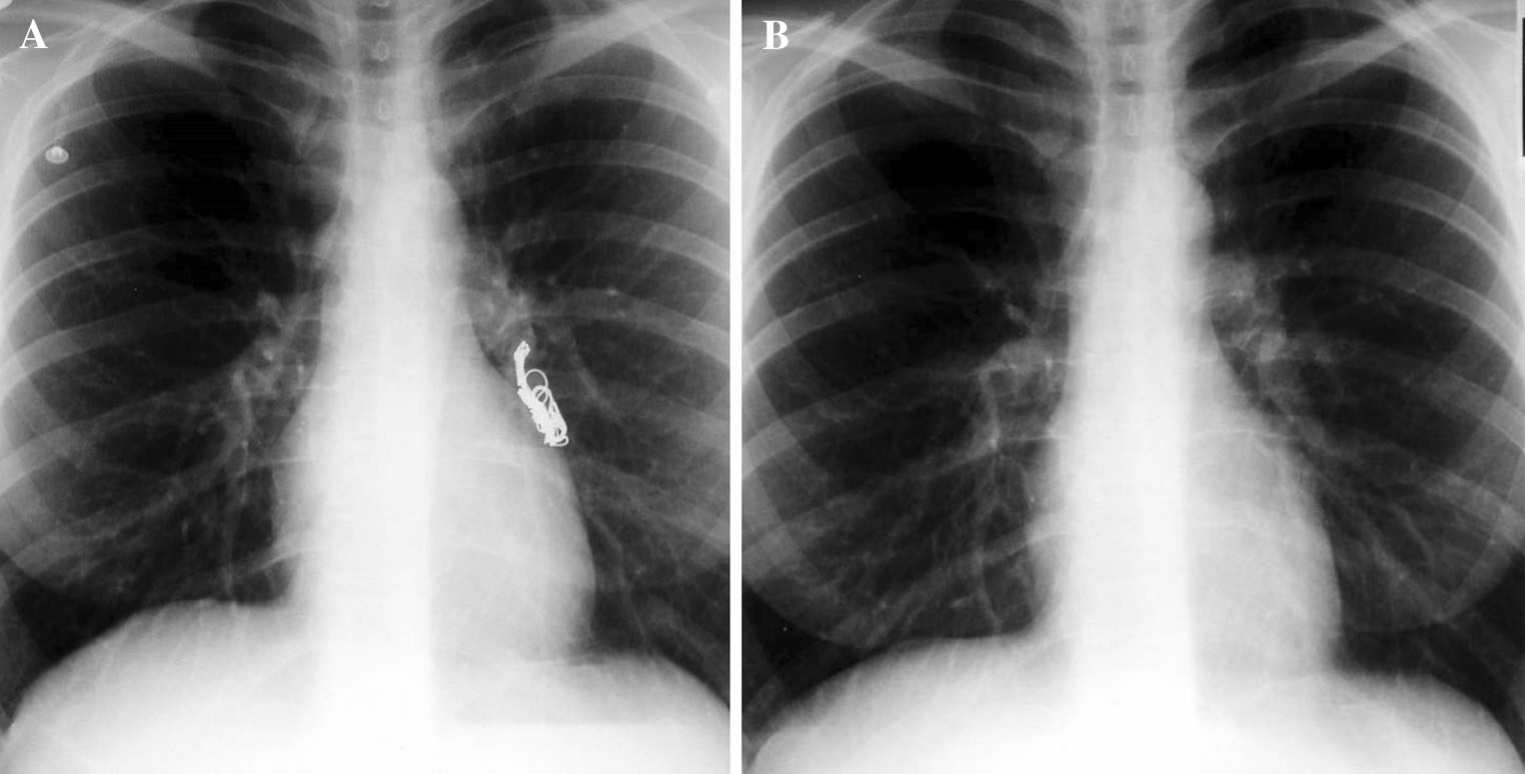
**A** and **B** Inadvertent pulmonary coil embolization (before and after removal)

- Enlarging pneumothorax, initially asymptomatic
- Coil migration—typically a post-procedural event, e.g. pulmonary embolization of coils (e.g. Fig. 15).

Such displaced and misplaced coils can be snared and retrieved relatively easily by means of conventional minimally invasive interventional radiology equipment and well-established techniques again using either the same jugular access or additional femoral venous access.

There is very little evidence to suggest that pelvic embolization is associated with an adverse effect on fertility associated with reduced ovarian ‘function’ [39] but this remains unknown. Indeed, although patients are advised to undergo the procedure when their family is complete, several patients have become pregnant and subsequently undergone successful confinement.

## Outcomes

Since Edward’s original case report of bilateral ovarian vein embolization in 1993 [16] to relieve pelvic pain, numerous studies have been reported initially with embolization of ovarian veins alone in relatively small numbers using coils [23, 40–42] or sclerosant alone [43] and subsequently of ovarian and internal iliac veins often in larger series using coils [21, 24], glue [25], sclerosant and coils together [4, 29–32]. The problem remains a lack of standardization of clinical assessment, imaging and treatment methods for two main conditions that are still not fully accepted, and the absence of consistently high level evidence by way of randomized control trial (RCT).

The earlier studies predominantly included women with PVC including pelvic pain assessed using a questionnaire or visual analogue scale (VAS). Partial or complete relief of chronic pelvic pain was achieved in up to 80 % of patients [29, 30] with relatively small patient numbers, whilst more recent studies treating both ovarian and internal iliac veins claim relief in up to 94 % in larger patient series [25]. Others have similarly had relatively large patient numbers but treated just ovarian vein reflux, such as Kwon who reports 82 % symptom improvement in 67 patients followed for up to 6 years from a review of medical records and telephone interviews [44]. Maleux et al., with a technical success of 98 % in 41 patients, followed up patients by questionnaire, reporting “total relief of symptoms in 59 % of patients with those undergoing unilateral compared to bilateral ovarian vein embolization [31].

Venbrux’s group [39] achieved 100 % technical success in 56 patients and recorded some degree of improvement (with a reduction of at least one point from baseline of the VAS score between baseline and follow up). Kim adopted a similarly aggressive approach embolizing all incompetent ovarian and internal iliac venous branches to eliminate all reflux [24]. This group evaluated the response of 127 patients with PVC over a 27–63-month duration again using questionnaires and a VAS, recording an improvement in 83 % over this relatively longer term follow up compared to previous studies. Furthermore, conservative management of patients with isolated internal iliac vein reflux did not resolve symptoms [21]. However, some reflux may occur in healthy subjects as valves are only found in approximately 10 % of internal iliac veins and their tributaries [45] and such as an aggressive strategy remains a matter of opinion.

Chung’s 2003 study [46] compared the efficacy of pelvic vein embolization with hysterectomy (with either unilateral or bilateral oophorectomy) for PVC associated non-cyclical chronic pelvic pain. Over a period of 4 years, 164 women were diagnosed with PVC “confirmed” with diagnostic laparoscopy and venography of ovarian and internal iliac veins. Of these patients, 118 failed to respond to medical therapy alone, and were recruited for randomization to embolotherapy and hysterectomy with either unilateral or bilateral oophorectomy. Pain was assessed with a VAS, and life changes in the previous 12 months analysed with a stress-scoring questionnaire used to subclassify into three groups. All patients underwent transfemoral venography, and Beard et al.’s pelvic venogram scoring system was used [47] in a modified form. Ovarian and internal iliac veins were variably embolized and embolotherapy was found to be significantly more effective than the other treatment arms in reducing pelvic pain, especially in patients with lower stress scores. One difficulty with this study is that it uses an infrequently adopted pelvic venous scoring system and a non-standardized protocol for coil placement was utilized. It is telling that it remains one of the only published RCTs to date in treating PVC syndrome with embolotherapy.

Later studies have concentrated on the more frequently occurring recurrent lower limb varicose vein patient group with either clinical assessment such as a decrease in venous clinical severity score [4] or using minimally invasive imaging such as transvaginal Doppler sonography [27].

Indeed, one of the largest series using follow-up Doppler assessment by just two vascular sonographers records well over 95 % success in ablating refluxing veins demonstrated pre-procedure following embolization by a single operator [32].

Monedero et al [26] evaluated 215 patients with recurrent varices after surgery (REVAS) with transvaginal Doppler sonography and selective pelvic venography. This group treated incompetent ovarian and internal iliac veins as well as collateral branches, with embolization using variably coils and foam using a transfemoral or basilic vein approach. Total relief of pelvic pain accompanied by reduced lower extremity venous stasis clinically was reported in 50 % of patients, and partial relief in 40 %. This group reported that the underlying cause of REVAS was partly due to blood leaking from the pelvis into the lower extremity.

A similarly large study by Ratnam et al. also looked specifically at the role of pelvic venous embolization in the management of lower limb varicosities [27]. 218 patients with pelvic venous incompetence as either a contributing factor or sole cause of varicose leg veins diagnosed exclusively with transvaginal duplex sonography were treated exclusively with platinum coil embolization. The study demonstrated a 100 % technical success rate using exclusively a transjugular approach with one reported coil misplacement without adverse effect [38] and two cases of pulmonary embolization of migrated coils, only one of which was symptomatic with the coils retrieved successfully using an endovenous approach.

A considerably smaller but more recent study also suggested that REVAS is reduced following embolization of incompetent pelvic veins and is particularly efficacious if patients are also experiencing symptoms of PVC [4] which often improve.

## Specific Considerations Managing Pelvic Venous Congestion Compared to Leg Varicosities of Pelvic Venous Origin

Clinically, it is clear that the spectrum of symptoms and physical signs in the two main patient groups with pelvic venous incompetence, i.e. PVC syndrome and lower limb varicose veins a priori or presenting as REVAS, is wide with considerable overlap. Radiologically, they are difficult to separate.

Empirically, investigators may think that PVC would be most often associated with incompetent ovarian, pudendal and other parametrial branches, whilst pelvic varices would be likely associated with refluxing obturator, circumflex and round ligament branches. However, the body of evidence suggests that it is not as simple as that. Scultetus described three clinical presentations in an overview of ‘female pelvic venous syndrome’—vulval varices without PVC symptoms, medial and posterior thigh varicosities associated with ovarian venous incompetence, and gluteal varicosities with vulval varices usually caused by internal iliac vein incompetence [48]. Experienced operators agree that the anatomy is so variable and pelvic venous incompetence so complex, and therefore, recommendations for treatment cannot be given [49].

## Discussion

For over 20 years, pelvic vein embolization (PVE) and most commonly ovarian vein embolization, has been performed principally for pelvic venous congestion syndrome (PVC). Evidence remains poor for its efficacy, and although initially anecdotal by way of case reports and small series, data is accumulating in larger series. There remains, however, a lack of robust evidence of its effectiveness, and this partly reflects the challenges of actually making the diagnosis clinically and radiologically, as well as the difficulty in assessing outcome. For PVC, symptomatic response is usually subjective but visual analogue scales (or variations thereof) have most often been used to attempt to identify a more objective outcome.

It may be argued that PVE is an established procedure that has been awaiting a significant clinical problem, and most interventional radiology suites do not perform the procedure in significant numbers. Increasingly, however, and over the last 10 years, more and more patients are being referred for the PVE, by vascular surgeons and other phlebologists, with pelvic venous reflux communicating with lower limb varicosities, and felt to be a significant or indeed the only cause of these veins.

Lower limb varicosities are typically associated with variable incompetence of the greater and lesser saphenous veins occasionally with perforating vein dysfunction [4, 38].

Although surgery is effective in eliminating or reducing lower limb varicose veins, recurrent varices after surgery (REVAS) ranges between 20 % and 80 % of patients between 5 and 20 years after initial surgery, depending on the definition of recurrence [4, 35, 38, 27, 50–54].

The commonest explanation of varicose vein recurrence in the lower limbs is neovascularization [55]. However, a more likely explanation is the development of collateral veins between the pelvis and lower limbs. Indeed, this is supported by growing evidence that primary or adjunctive treatment by PVE allows for the successful treatment of symptomatic lower limb venous recurrences [43]. Such a collateral network of veins has previously been described [56] and may be demonstrated by catheter venography with incompetence confirmed by ‘functional’ duplex sonography. It has also been reported that no more than 45 % of these recurrences occur in the region of the greater saphenous vein suggesting reflux from pelvic veins as the cause [57].

Furthermore, many studies have demonstrated lower limb varicosities partly or entirely of pelvic venous origin with the latter group having a competent saphenofemoral junction [58–63] and not confined to the perineal, gluteal or perianal tissues.

Minimally invasive imaging methods such as selective catheter venography [17] and non-invasive studies including duplex sonography [3, 5, 6, 64–66], computed tomography (CT) [67, 68] and magnetic resonance imaging (MRI) [69, 70] are most frequently used to assess pelvic and lower limb venous incompetence.

Selective venography has been considered the gold standard for demonstrating pelvic incompetence as it can potentially show communication between incompetent pelvic veins (ovarian and/or internal iliac) and lower limb varicosities, but a recent study cautions reliance on this modality [71]. However, as dilated and presumed incompetent veins have been described in asymptomatic parous women [2], a more functional assessment of pelvic venous incompetence may be required and transvaginal, transperineal or transrectal duplex sonography have been suggested to be such an investigation [5, 6]. Pre-operative assessment with functional imaging techniques not involving ionizing radiation such as ultrasound or MR venography in an ‘upright scanner’, is desirable and such techniques may be reproducible with appropriate training.

Although ultrasound is well suited to ‘direct’ therapeutic embolization to minimize financial and radiation ‘costs’ associated with unnecessarily embolizing non-refluxing veins, it is highly operator dependent. One advantage is its use in assessing the outcome of therapeutic embolotherapy in determining whether the procedure has been ‘successful’ in eliminating diagnosed reflux and identifying any new reflux prior to definitive ‘pinhole’ endovenous lower limb surgery.

Endovenous ablation of pelvic venous incompetence is a relatively simple, safe and efficacious technique for occluding and obliterating refluxing veins associated with pelvic venous congestion syndrome or implicated in the aetiology of de novo or recurrent lower limb venous varicosities with or without vulval or perivulvar veins. Conventional catheter techniques are used typically under conscious sedation employing commonly available occlusive agents including liquid sclerosants, glue, foam and most commonly platinum coils. The latter are highly visible and do not preclude patients undergoing future MRI imaging (although artefact is created on CT scanning). The procedure has a shallow learning curve and complications associated with therapeutic embolization are well defined, rare and easily managed percutaneously.
